# Impact of an Asynchronous Spaced Education Learning Intervention on Emergency Medicine Clinician Opioid Prescribing

**DOI:** 10.7759/cureus.18165

**Published:** 2021-09-21

**Authors:** Tyler W Barrett, Matt D McEvoy, Leslie C Fowler, Matthew S Shotwell, Yaping Shi, Michael Costello, Devin Rogers, Jennifer Slayton, David A Edwards

**Affiliations:** 1 Department of Emergency Medicine, Vanderbilt University Medical Center, Nashville, USA; 2 Department of Anesthesiology, Vanderbilt University Medical Center, Nashville, USA; 3 Department of Biostatistics, Vanderbilt University School of Medicine, Nashville, USA; 4 Department of Quality, Safety and Risk Prevention, Vanderbilt University Medical Center, Nashville, USA; 5 Department of Emergency Medicine, PeaceHealth Sacred Heart Medical Center University District, Eugene, USA

**Keywords:** prescribing, narcotic, acute pain, emergency medicine, education, opioid epidemic

## Abstract

Introduction

Opioid prescribing has contributed to the opioid crisis and education has focused on improved opioid stewardship. We aimed to evaluate the impact of an asynchronous high-quality education to change emergency medicine (EM) clinician opioid prescribing.

Methods

We conducted a retrospective cohort study of a spaced-education intervention in EM clinicians who work at an urban, university-affiliated academic medical center emergency department. We developed opioid prescribing educational content and investigated whether prescriber participation in a novel asynchronous educational program, QuizTime, was associated with a change in EM clinician opioid prescribing practices and whether those prescribing practice changes would be maintained. The primary outcome was the frequency of opioid prescriptions by attributable emergency department discharges. We compared the frequency during the post-intervention period, 24 months following QuizTime education (July 2018 - June 2020) to the baseline period (November 2016 - March 2018). The secondary outcomes were total morphine milligram equivalent (MME) and the number of tablets dispensed per prescription. We analyzed the outcomes by EM clinicians’ level of participation in QuizTime education.

Results

During the study period, there was an overall reduction in opioid prescribing per attributable emergency department discharge (p < 0.001). Among the 45 prescribers who enrolled in QuizTime, there was a significant reduction of 4.3 (95% CI: 3.9, 4.6, p < 0.001) opioid prescriptions per 100 ED discharges in the post-intervention period compared to baseline. Among the 11 non-enrollees, there was a significant reduction of 2.4 (95% CI: 1.7, 3.1, p < 0.001) opioid prescriptions per 100 emergency department discharges in the post-intervention period compared to baseline. The prescribers enrolled in QuizTime had a significantly larger reduction in prescriptions compared to those who did not enroll (p < 0.001). A decreasing trend of total MME and the number of tablets dispensed was observed (p < 0.001). However, there was insufficient evidence to show a reduction in the number of tablets dispensed or MME per day.

Conclusion

EM clinician participation in the QuizTime Pain Management educational program was associated with a nearly two-fold decrease in opioid prescriptions per emergency department discharge compared to peers who chose not to enroll.

## Introduction

As the United States continues to cope with the opioid epidemic, there has been an increased emphasis on incorporating non-opioid alternatives for pain management in the emergency department (ED) [1-7]. Emergency medicine has actively worked toward reducing the use of opioids in the ED for treatment of acute pain and decreasing the quantity of opioids prescribed [2-4]. Educating clinicians on the dynamic field of pain management and the rapidly evolving federal and state regulations related to opioid prescribing is a critical element of practice change.

In 2018, Tennessee enacted a comprehensive plan to end the state’s opioid epidemic and on July 1, 2018, legislation went into effect imposing multiple new requirements for opioid prescribing [8]. The requirements included categorization of patients by the reason for pain needing treatment with opioids (e.g., acute pain requiring less than three days of opioid treatment, pain after surgery, or one of several conditions deemed exempt). The law also required obtaining written informed consent prior to prescribing and inclusion of ICD-10 diagnosis codes with each prescription [8]. This 2018 law was then modified during a subsequent legislative session resulting in a revised law that prescribers and pharmacists had to learn. Educating prescribing clinicians in Tennessee was an important obligation for many hospitals and teaching institutions statewide [8].

Despite the proliferation of online and in-person educational opportunities for controlled substance continuing medical education (CME), the dynamic nature of the opioid epidemic and the constantly changing legislative landscape necessitated an adaptable educational strategy more easily disseminated to a large group of prescribers across the state. We investigated whether a novel educational program, QuizTime, might effectively fulfill the educational obligation as measured by practice change among emergency medicine (EM) clinicians. QuizTime, an asynchronous learning paradigm developed at Vanderbilt University Medical Center (VUMC) for educators and learners, has been used to educate multiple learner groups in pain management [9]. We aimed to measure whether participation in the QuizTime Pain Management educational program was associated with EM clinician opioid prescribing practices and whether those prescribing practice changes would be maintained.

## Materials and methods

This study was performed at VUMC, an urban, university-affiliated, level 1 trauma center, that treats approximately 70,000 adult patients annually. The VUMC institutional review board approved this study designed as a retrospective cohort comparing the prescribing differences among EM clinicians that participated versus those who did not participate in an educational intervention. The target population was EM clinicians working at VUMC during the entire study period from November 1, 2016, through June 30, 2020. The EM team included 63 faculty attending physicians and four advanced practice providers (APPs). All EM clinicians were invited to participate in the study during meetings or by email four weeks prior to the study start date. Interested participants completed a short web-based survey that collected their name, continuing medical education (CME) user identification, email address, and cell phone. EM clinicians received occasional pain treatment-related education via email or didactic lectures throughout the calendar year irrespective of their participation in the QuizTime study.

QuizTime educational intervention

The QuizTime (Center for Advancing Mobile Health Learning, CAMHL, Nashville, TN) educational intervention was designed to inform clinicians on responsible opioid prescribing. The intervention included 20 multiple choice questions delivered to the participants, one each weekday for 20 days beginning on the date of their enrollment during the enrollment period of April 1 and June 30, 2018. This format is known as spaced education; education that is delivered with topical overlap and some repeated information in a testing learning format to increase topic-specific knowledge [10]. The question items covered a broad range of topics related to evidence-based pain management and opioid stewardship. They were designed to educate clinicians by modeling best practices through case studies. The questions were written and edited by an expert panel of trained question writers with expertise in acute and chronic pain management and additionally reviewed by emergency medicine physicians to ensure relevance to the practice of emergency medicine. Additionally, a structured process and format were followed for creating each question item so that practice take-home points were easily digested, and background explanation material was available for more thorough understanding. Study participants chose whether to receive the questions by email or text message to their phones. The clinicians had 24 hours to answer each question before the question would expire but continued to have access to the answer and educational material for ongoing reference. Participants engaged with the QuizTime educational intervention by answering the multiple-choice question right or wrong. After answering, the correct answer was displayed along with a detailed rationale including key references (example provided in the Appendix). The quiz was accredited for one credit hour of pain-management-specific continuing medical education (CME) for every four answered questions. The QuizTime software electronically stored the participants’ names, selected answers, percent answered correctly, and the number of questions attempted during the study period. Additionally, we recorded the number of CME credit hours awarded as an indirect measure of participant engagement.

Pain medication administration tracking

Opioid administration and prescribing data were collected for the entire duration of the study. For each participant, we collected data for a total of 44 months: 17 months prior to the intervention (baseline period), a three-month period during which the four weeks long QuizTime intervention was administered (intervention period), and 24 months after the conclusion of the education (post-intervention period). Extended baseline and post-intervention periods were obtained to control for co-occurring events that may also have been associated with prescribing trends during the same time period (e.g., changes were made to the setting defaults for opioids on November 2, 2017), with migration to a new electronic health record (EHR), (Epic, Verona, WI), and this association was reported on previously [11].

Opioid prescribing source database

The Vanderbilt Committee on Opioid Monitoring and Stewardship (VCOMS) is a multidisciplinary committee consisting of representatives from the institution’s departments of anesthesiology, emergency medicine, hospital administration, pharmacy, and quality, safety, and risk prevention. VCOMS maintains a database of provider prescribing and provided the dataset for this study. The feature set included provider details, prescribing location, prescription details for opioid and non-opioid pain medications, morphine milligram equivalent (MME) conversions of daily opioid prescriptions.

Outcome measures

The primary outcome was the frequency of opioid prescriptions by attributable ED discharges. Secondary outcomes included MME per day per opioid prescription, the number of pills dispensed per prescription, and the total MME per opioid prescription. An EM attending sees all patients during their shift and determines the discharge prescriptions. An EM attending’s engagement was measured by the number of QuizTime CME hours awarded per individual.

Statistical analyses

Attending demographic, baseline characteristics, and prescription outcomes were summarized using descriptive statistics, including the median and interquartile range (IQR) and mean +/- standard deviation for quantitative variables, and percentages for categorical variables. Attendings who enrolled in QuizTime were analyzed with the QuizTime participation group regardless of the number of questions answered or CME credits awarded. For the breakdown of opioid prescribing by training, data were available only for the period after hospital transition to a new electronic medical record. Summaries were stratified by baseline period (November 2016 - October 2017), Epic EHR pre-intervention period (November 2017 - June 2018), and Epic post-intervention period (July 2018 - June 2020). Nonparametric Wilcoxon rank-sum test and the Pearson Chi-square test were used for comparisons as appropriate.

The linear mixed-effect model was used to examine the effect of the QuizTime intervention on opioid prescribing patterns including total MME per opioid prescription, MME per day per prescription, and the number of pills dispensed per prescription. Attending physician effect was treated as a random effect in the model to take care of the provider’s heterogeneity. The time trend was modeled using a flexible regression technique (e.g., restricted cubic splines) interacting with co-occurring events, the migration to a new electronic medical record in November 2017, QuizTime from April to June 2018, and the Tennessee legislation enacted on July 1, 2018. Outcome variables were log-transformed for their right-skewed distributions. Effect sizes were presented with 95% confidence intervals. Hypothesis testing was implemented using Wald-type methods with a type I error rate of 5%. All statistical analyses were implemented using the statistical software package R and the relevant add-on packages.

## Results

Of the 67 attending EM clinicians and APPs invited to participate in the pilot, 11 were excluded due to no longer working at our institution as of July 1, 2020, leaving 56 attendings and APPs comprising the cohort. Of those, 45 (74%) enrolled in the course and were on staff in the ED for the duration of the study period. Two of the 45 clinicians did not answer any of the 40 questions and received 0 CME credits. An additional 11 did not answer at least four questions to meet the minimum required for CME credit. Table 1 presents additional details on the clinician prescriber population.

**Table 1:** Distribution of emergency medicine prescribers (n = 56) *Two clinicians enrolled in QuizTime but did not answer any questions.

Variable	Participated in QuizTime	Did not participate in QuizTime
Total Prescribers	45 (80.4%)*	11 (19.6%)
Female	12 (26.7%)	7 (63.6%)
Years in Practice (median (IQR))	11 (7, 16)	7 (3, 18)

Between November 2016 and June 30, 2020, there were 9427 (7.0%) opioid prescriptions written among the 134,613 patients discharged from the ED. Ordering prescribers included resident physicians (51.3%), attending physicians (40.6%), and APPs (8.1%). During the study period, there was an overall reduction in opioid prescribing per attributable ED discharge. Among the 45 prescribers who enrolled in QuizTime, there was a significant reduction of 4.3 (95% CI: 3.9, 4.6, p < 0.001) opioid prescriptions per 100 ED discharges in the post-intervention period compared to baseline. Among the 11 non-enrollees, there was a significant reduction of 2.4 (95% CI: 1.7, 3.1, p < 0.001) opioid prescriptions per 100 ED discharges in the post-intervention period compared to baseline. The prescribers enrolled in QuizTime had a significantly larger reduction in prescriptions compared to those who did not enroll (p < 0.001).

The median total MME per prescription reduced from 68 to 60 during the study period and was similar among clinicians who did and did not participate in the educational intervention (Table 2).

**Table 2:** Distribution of prescriptions (baseline and post-QuizTime intervention) among prescribers who participated in QuizTime Continuous variables presented as Median and interquartile range (IQR). Dichotomous variables presented as frequency and percentage with 95% confidence intervals (95% CI). The estimated attributable ED discharges were calculated by multiplying the percentage of attendings who enrolled in QuizTime by the total number of ED discharges over that time interval. MME: Morphine milligram equivalent; APP: Advanced practice provider.

Variable	Baseline pre-Epic period	Post-intervention period
Total Months, n	17	24
Total Opioid Prescriptions	4459	2981
Estimated Attributable ED Discharges	45,828	54,782
Opioid Prescriptions/Attributable ED Discharge (%)	9.7% (95% CI: 9.5, 10.0)	5.4% (95% CI: 5.3, 5.6)
Ordering Provider		
Attending physician	Not available	1586 (53.2%)
Resident physician	Not available	1138 (38.1%)
APP	Not available	142 (4.7%)
Dispense Quantity	12 (9, 12)	9 (8, 12)
Total MME	68 (60, 100)	60 (45, 90)

**Figure 1:**
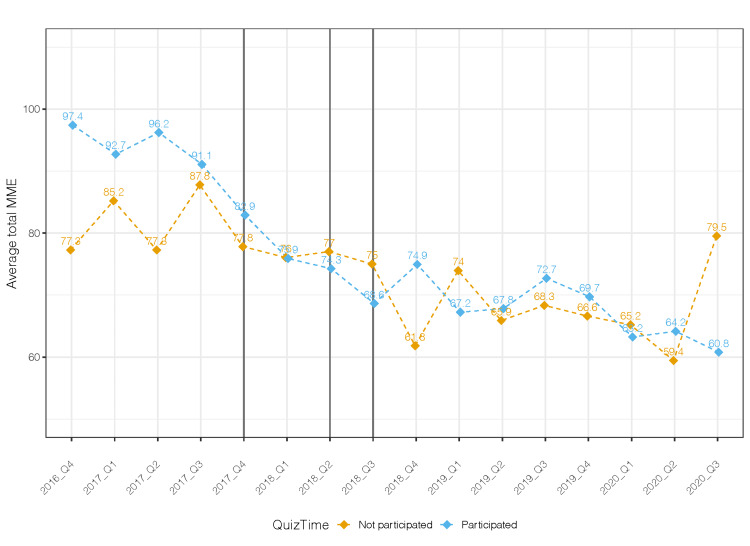
Quarterly average total MME by the intervention MME: Morphine milligram equivalent

There is a greater decline in the total MME per prescription among individuals who earned one or more QuizTime educational credits compared to those who earned no credits or did not participate (Figure 1).

There was a significant decreasing trend over time in total MME after controlling for provider effect. Compared to baseline, both those who participated and did not participate had a significant reduction in total MME during the post-intervention period after controlling for time and provider effect (p = 0.004, p = 0.01). However, there was no significant difference in total MME between those who did and did not participate in QuizTime during the post-intervention period. The number of pills per prescription decreased from a median of 12 (IQR: 9, 12) during the baseline period to 9 (IQR: 8, 12) during the post-intervention period in the participating group (Table 2) and in the nonparticipating group (Table 3).

**Table 3:** Distribution of prescriptions (baseline and post-QuizTime intervention) among prescribers who did not participate in QuizTime Continuous variables presented as Median and interquartile range (IQR). Dichotomous variables presented as frequency and percentage with 95% confidence intervals (95% CI). The estimated attributable ED discharges were calculated by multiplying the percentage of attendings who enrolled in QuizTime by the total number of ED discharges over that time interval. MME: Morphine milligram equivalent; APP: Advanced practice provider.

Variable	Baseline	Post-Intervention
Total Months, n	17	24
Total Opioid Prescriptions	1049	938
Estimated Attributable ED Discharges	11,172	13,391
Opioid Prescriptions/Attributable ED Discharge (%)	9.4% (95% CI: 8.9, 9.9)	7.0% (95% CI: 6.6, 7.5)
Ordering Provider		
Attending	Not available	330 (35.2%)
Resident	Not available	517 (55.1%)
APP	Not available	51 (5.4%)
Dispense Quantity	12 (9, 12)	9 (8, 12)
Total MME	68 (50, 90)	60 (45, 90)

There was a significant decreasing trend over time in the number of opioid tablets dispensed (p < 0.001). However, there was no sufficient evidence showing differences in dispense between baseline versus post-intervention after controlling for time and provider effect. The median MME dose per day during the baseline period was the same between participating and non-participating groups at 22 (IQR: 20, 30). Compared to baseline, neither group demonstrated a significant change in the post-intervention period.

Based on the exploratory analysis, the transition to the Epic EHR was associated with a reduction in opioid prescription patterns. Before the Epic transition (i.e., baseline), the participating group had a total MME median of 68 (IQR: 60,100) and after the Epic transition was reduced to 60 (IQR: 45,90) (p = 0.004), and that remained at 60 (IQR: 45,90) during the post-intervention period (p = 0.07). Among those who did not participate, the baseline median MME per prescription was 68 (IQR: 50, 90) and after the Epic transition the median decreased to 60 (IQR: 55, 90) (p = 0.01), and then remained at 60 (IQR: 45, 90) during the post-intervention period (p = 0.22).

**Figure 2:**
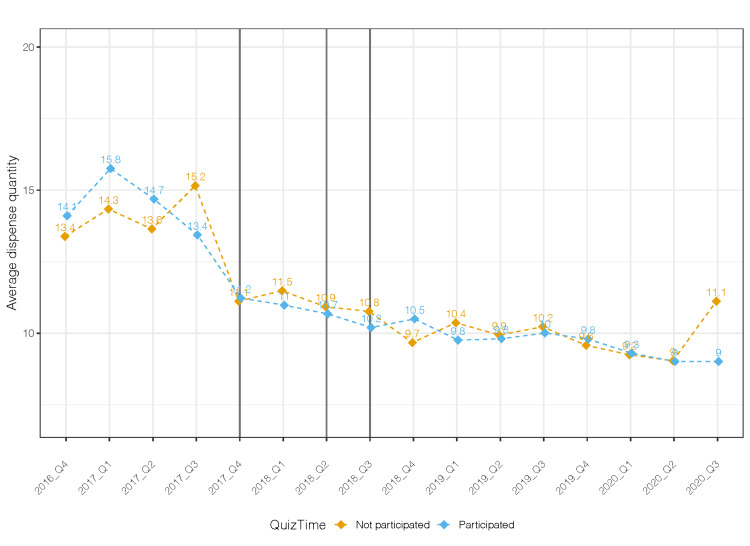
Quarterly average dispense quantity by the intervention (Participated in QuizTime, n = 45; Did not participate in QuizTime, n = 11)

When stratifying by engagement, similar results were observed among the three categories (earned 0 CME credits, 1-5 CME credits, and 5-8 credits). All categories had a significant reduction in total MME per prescription in the period following Epic transition compared to the baseline, but no further reduction after participating in the QuizTime education. There was a downward trend in the total number of pills prescribed among those who participated in QuizTime (Figure 2).

## Discussion

The opioid epidemic in the United States and its associated morbidity and mortality has focused much public health and legislative attention on reducing the number of opioid tablets in the community. Identifying novel approaches to educate prescribers about new prescribing requirements across a municipality, let alone an entire state, is a challenge. We investigated whether EM clinician participation in the QuizTime Pain Management educational program was associated with a change in opioid prescribing practices and whether those prescribing practice changes would be maintained. Overall, we identified a decline in the number of opioid prescriptions per attributable ED discharge with a larger reduction in the group that enrolled in the QuizTime course, demonstrating that EM clinicians were less likely to discharge a person with an opioid prescription. During the baseline period, there was an apparent increase in the frequency of opioid prescriptions around the time of our EHR transition. While this can’t be entirely explained, one possibility is differences in data capture between legacy and new systems. Another potential explanation is the possibility that prescribers, anticipating the impending change in law, were more likely to prescribe.

The study identified a decreasing trend in the total MME per prescription. A core concept within the QuizTime opioid education material is to prescribe the lowest effective dose opioid for the shortest duration necessary to manage pain not adequately controlled with non-opioid multimodal medications and treatments. However, when looking just at those patients who received a prescription, we did not identify a significant difference in the prescribing practices among those who enrolled in QuizTime. In other words, when opioids were prescribed, the doses were not appreciably different despite the educational materials emphasizing the need to reduce the dose and duration of prescriptions. A possible explanation for this is that those interested in participating in QuizTime education already felt they were prescribing appropriately.

Our analysis did show a continued decline in the total MMEs and number of pills prescribed by our clinicians for two years after the state prescribing legislation was enacted. Possible reasons for the continued decline include our medical center’s focus on opioid stewardship, continued attention to the opioid epidemic in medical specialty literature and press, and the increasing number of patients presenting to EDs with complications related to opioid use disorder.

This study’s results should be understood in the context of the following limitations and potential biases. First, interested clinicians were asked to participate and voluntary enrollment may bias towards participants who feel they are practicing well, confirming their practice, or who may hope to learn and be more likely to change. Second, clinicians’ knowledge and prescribing practices related to opioids may have been influenced by other means including the increased national and local attention to opioid use. Third, as an academic medical center, prescriptions written by resident physicians are attributed to the supervising attending, and while the attending signs off on patient care, there may be situations where a resident physician prescribed a quantity, strength, or duration of an opioid that was not consistent with what the emergency attending would prescribe in their own practice. Thus, the QuizTime education might have been more effective in a setting where prescriptions are more directly attributable to the practice patterns of the person signing the prescription, such as in a private practice setting. Fourth, during the analysis, we noted a significant reduction in prescribing during the baseline period with the implementation of a new EHR and changes in the order set for opioids. A decrease in prescription dose during the baseline period may have diluted the measurable change due to QuizTime education. The new EHR did not include best practice advisories related to opioid prescribing or pain management during the study period. Finally, the national focus on the opioid epidemic and continued focus within our medical center may have also impacted prescribing practices; however, such external forces would be expected to influence both prescribers who participated in the QuizTime education and those who did not enroll.

## Conclusions

In conclusion, EM clinician participation in the QuizTime Pain Management educational program was associated with a nearly two-fold decrease in opioid prescriptions per ED discharge compared to peers who chose not to enroll. The strength of this study is in the quality of the education materials and the delivery method, optimized to promote durable change in practice. The potential impact may have been attenuated by changes to the EHR prior to the intervention and prescribing processes unique to an academic teaching hospital. Future research should consider the relative impact of targeted education, institutional process change, and legislation in promoting practice change among prescribers.

## Figures and Tables

**Figure 3:**
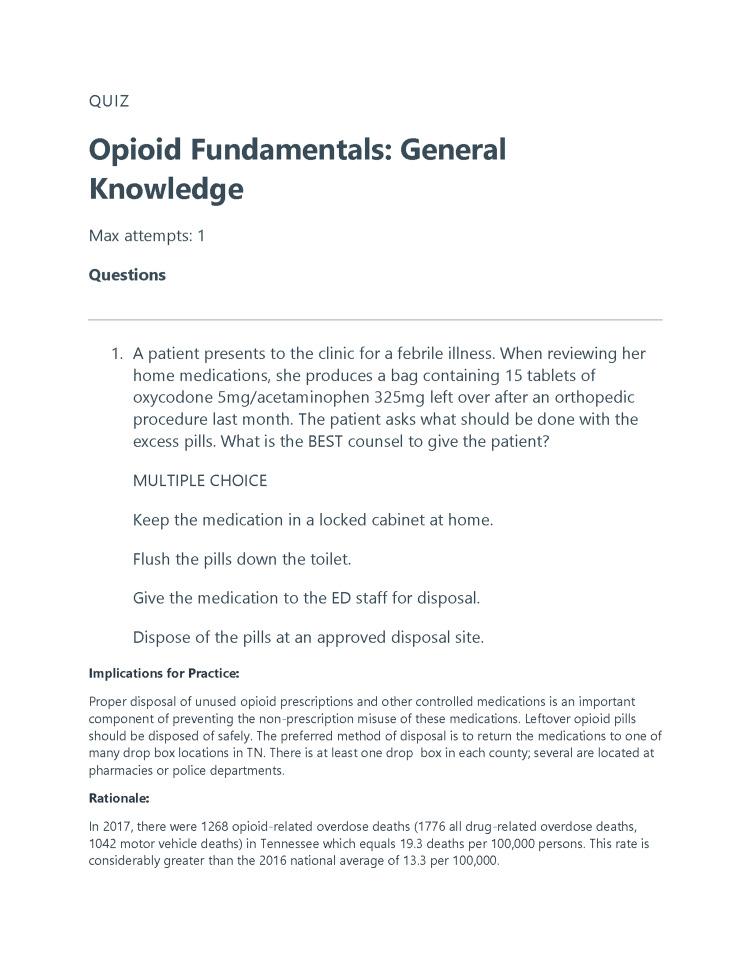
Example of QuizTime educational tool
